# Dual Role of *CACNA1C*/Ca_V_1.2 in Cardiac and Neuropsychiatric Disease

**DOI:** 10.3390/biom16101459

**Published:** 2026-10-07

**Authors:** David Königstein, Theresa M. Kisko, Susanne Michels, Judith Alferink, Markus Wöhr, Carsten Culmsee, Jens Kockskämper

**Affiliations:** 1 Institute of Pharmacology and Clinical Pharmacy, Faculty of Pharmacy, Marburg University, Karl-von-Frisch-Str. 2 K|03, 35043 Marburg, Germany; david.koenigstein@staff.uni-marburg.de (D.K.); culmsee@staff.uni-marburg.de (C.C.); 2 Social and Affective Neuroscience Research Group, Laboratory of Biological Psychology, Research Unit Brain and Cognition, Faculty of Psychology and Educational Sciences, KU Leuven, 3000 Leuven, Belgium; theresa.kisko@kuleuven.be (T.M.K.); markus.wohr@kuleuven.be (M.W.); 3 Leuven Brain Institute, KU Leuven, 3000 Leuven, Belgium; 4 A.I.Virtanen Institute for Molecular Sciences, University of Eastern Finland, 70211 Kuopio, Finland; 5 Institute for Biomedicine, Eurac Research, 39100 Bolzano, Italy; 6 Department of Psychiatry, University of Münster, 48149 Münster, Germany; judith.alferink@ukmuenster.de; 7 Cells in Motion Interfaculty Cluster, University of Münster, 48149 Münster, Germany; 8 Social and Affective Neuroscience, Experimental and Biological Psychology, Faculty of Psychology, Marburg University, 35037 Marburg, Germany; 9 Center for Mind, Brain and Behavior (CMBB), Marburg University, 35032 Marburg, Germany

**Keywords:** *CACNA1C*, Ca_V_1.2, L-type calcium channel, cardiomyocyte, neuron, glia, heart, brain, disease, Timothy syndrome, calcium channel blockers

## Abstract

The *CACNA1C* gene encodes the α_1C_ subunit of voltage-gated Ca_V_1.2 calcium channels, serving many pivotal functions throughout the body, including in the heart and brain. Clinical and genetic evidence implicates altered expression and/or function of *CACNA1C*/Ca_V_1.2 in both cardiac and neuropsychiatric disease, including hypertension, cardiac arrhythmias, coronary artery disease, schizophrenia, bipolar disorder, depression, and autism. Gain-of-function mutations in *CACNA1C* cause Timothy syndrome, a potentially lethal multi-organ disorder with severe cardiac and neuropsychiatric defects. Conversely, the complete loss of *Cacna1c* is lethal in mice. The development of viable mouse and rat models with *Cacna1c* haploinsufficiency has allowed us to study the consequences of reduced expression and function of *Cacna1c*/Ca_V_1.2 for heart and brain function from the molecular and cellular to the behavioral level. We review recent findings from these rodent models, revealing (i) alterations in behavior and (ii) in neuronal and non-neuronal function, as well as (iii) an impaired ability of the heart and its cardiomyocytes to adapt to (sympathetic) stress. Results lend further support to the notion that *CACNA1C*/Ca_V_1.2 dysfunction represents a common link between cardiac and neuropsychiatric disorders and that appropriately targeting *CACNA1C*/Ca_V_1.2 in selected patients may hold the promise of treating both conditions effectively with a single drug.

## 1. *CACNA1C*/Ca_V_1.2 in Physiology and Pathology

**Figure 1:**
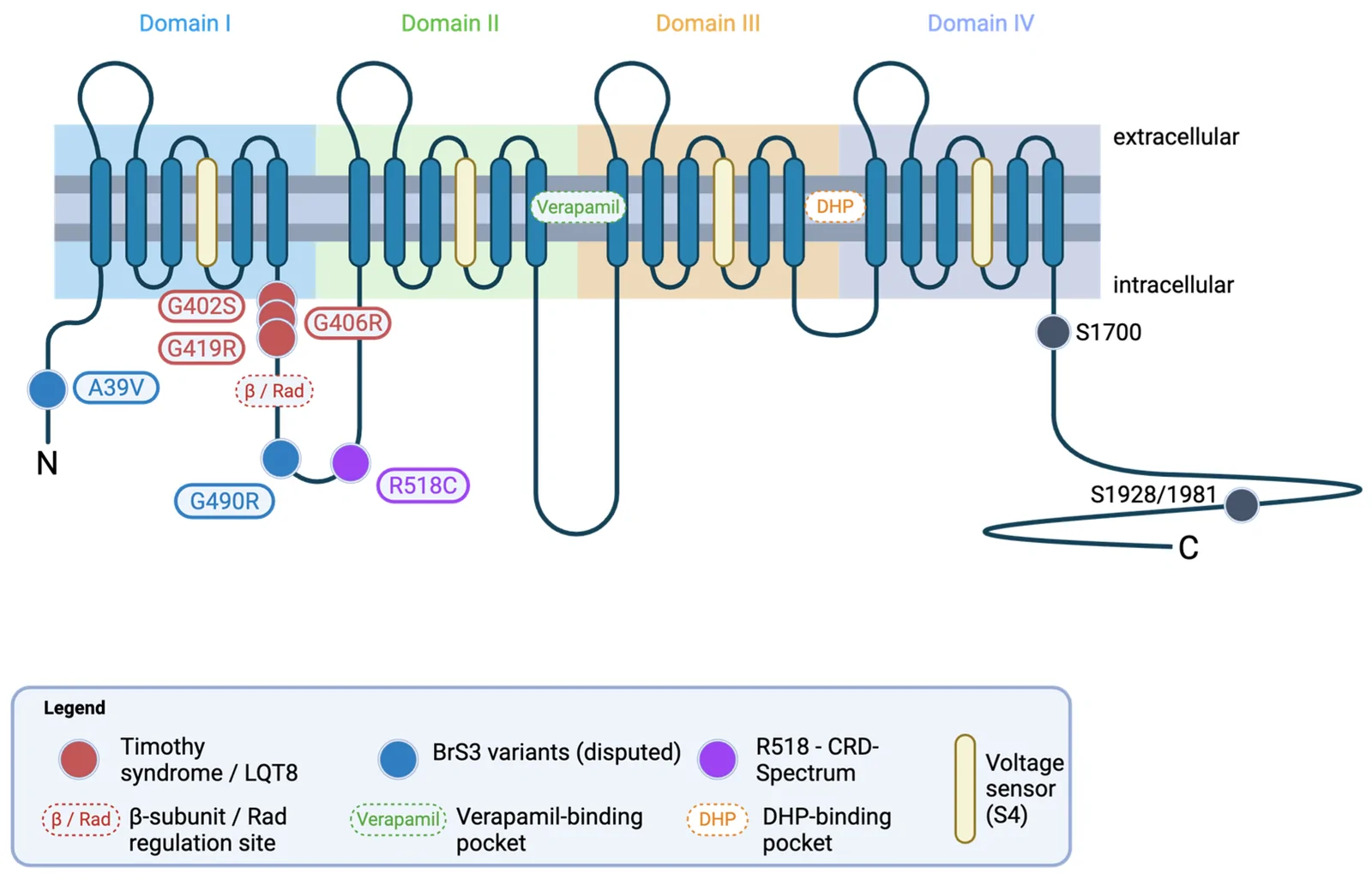
Domain structure and membrane topology of Ca_V_1.2. Ca_V_1.2 is a single integral membrane protein consisting of four domains (I–IV) with six transmembrane segments (S1–S6) each. The S4 voltage sensors are highlighted in yellow. Cryo-EM structures of human Ca_V_1.2 in apo and ligand-bound conformations [8,9] have resolved the major drug-binding pockets: Dihydropyridines (DHP) bind a III–IV fenestration on the lipid-facing surface. Verapamil and other phenylalkylamines occlude the inner pore from the cytoplasmic side; this binding mode has been characterized in Ca_V_1.1 cryo-EM structures and modeled for Ca_V_1.2 by homology [10]. Variant mapping onto this schematic places gain-of-function variants (red; G406R, G402S, G419R) predominantly in proximity to the S6 segment of domain I (the activation gate); loss-of-function variants (blue; A39V, G490R) localize to the cytoplasmic N-terminal or I–II linker regions; the mixed variant R518C (purple) sits in the I–II linker. The β-subunit/Rad interaction site on the I-II linker mediates β-adrenergic regulation; two candidate PKA phosphorylation sites on the C-terminal tail are indicated: the proximal S1700 site and the distal S1928/S1981 site. Created in BioRender. Königstein, D. (2026) https://BioRender.com/w0fm4tk.

The *CACNA1C* gene, located on chromosome 12p13.33, encodes the pore-forming α_1C_ subunit of the voltage-gated L-type calcium channel Ca_V_1.2. Functional Ca_V_1.2 channels are heteromeric protein complexes composed of the pore-forming α_1C_ subunit together with auxiliary β and α2δ subunits and constitutively associated calmodulin (CaM) molecules, which are required for functional expression and cell surface trafficking [1,2]. The major channel characteristics including voltage sensor (S4 segments), ion selectivity, and pharmacological drug-binding sites reside in the α_1C_ subunit, which consists of four domains (I-IV) with six transmembrane segments each (Figure 1). Similar to other L-type channels, Ca_V_1.2 generates long-lasting calcium currents in response to strong membrane depolarization and is sensitive to blockade by dihydropyridines (DHP, such as nifedipine), phenylalkylamines (such as verapamil) and benzothiazepines (such as diltiazem) [3].

Ca_V_1.2 is expressed throughout the human body and it is involved in the regulation of a multitude of pivotal processes. For example, Ca_V_1.2 is found (1) in neurons in the nervous system, where it regulates various aspects of neuronal activity including gene expression and synaptic plasticity; (2) in cardiomyocytes in the heart, where it is critically involved in the regulation of heart rate, atrioventricular (AV) conduction and cardiac contractility; (3) in arterial smooth muscle cells, where it regulates arterial diameter and, hence, tissue perfusion and blood pressure; (4) in intestinal smooth muscle cells, where it regulates motility; or (5) in hormone-producing cells, like β-cells of the pancreas or chromaffin cells of the adrenal medulla, where it contributes to hormone secretion [3,4]. Not surprisingly, therefore, complete (homozygous) loss of *Cacna1c* is lethal in mice before embryonic day 14.5 [5]. Moreover, cardiomyocyte-restricted homozygous deletion of *Cacna1c* in adult mice is lethal within less than two weeks owing to a massive loss of cardiac contractility [6]. On the other hand, a gain-of-function mutation in Ca_V_1.2 causes Timothy syndrome (TS), a severe multi-organ disorder characterized by cardiac and neuropsychiatric abnormalities including lethal cardiac arrhythmias, congenital heart disease, cognitive abnormalities and autism, but also by immune deficiency and hypoglycemia [7]. Thus, mammalian life is not feasible without Ca_V_1.2 and major alterations in its expression or function cause severe disease that affects various organ systems, most of all heart and brain.

## 2. *CACNA1C*/Ca_V_1.2 in Neuropsychiatric Disorders and Behavior

### 2.1. Genetic Evidence Implicating CACNA1C in Neuropsychiatric Disorders

The first evidence linking Ca_V_1.2 to neuropsychiatric phenotypes emerged from investigations of TS, a rare multisystem disorder resulting from de novo gain-of-function mutations in *CACNA1C* [7]. TS is characterized by cardiac arrhythmias and a distinct neuropsychiatric phenotype including cognitive abnormalities and autism. The exonic G406R missense mutation (Figure 1) leads to increased Ca_V_1.2 channel opening at subthreshold potentials as well as reduced voltage-dependent inactivation resulting in elevated calcium entry. Since its first introduction, the spectrum of phenotypes in TS has expanded to include more variants and affected organ systems. The high variability in the expression of *CACNA1C* and the resultant physical, physiological and behavioral outcomes has led to a call for a standardized definition for individuals presenting with pathogenic variants that may be linked to *CACNA1C* [11,12].

In addition to rare coding variants, genome-wide association studies (GWAS) have identified multiple common neuropsychiatric risk-associated single-nucleotide polymorphisms (SNPs) with a substantial genetic overlap between different diagnoses. Over the past two decades, *CACNA1C* has emerged as one of the most robust and reproducible risk loci across various neuropsychiatric disorders including schizophrenia, bipolar disorder, major depressive disorder, attention deficit-hyperactivity disorder, and autism spectrum disorder [13]. Several psychiatric risk-associated SNPs have been found within non-coding regions of *CACNA1C*, most prominently the intronic variant rs1006737 (risk allele A, global minor allele frequency 0.30, odds ratio ~1.15) [14,15]. These variants are enriched in intron 3, which harbors regulatory elements that physically interact with the *CACNA1C* promoter through chromatin looping. Neuropsychiatric risk alleles within this region disrupted enhancer–promoter communication, thereby decreasing transcriptional activity and reducing *CACNA1C* mRNA expression [16]. Functional studies of rs1006737 report heterogeneous effects on *CACNA1C* mRNA abundance, with evidence for both up- and downregulation [17,18,19]. This variability likely reflects context-dependent regulation influenced by developmental stage, brain region, cellular identity, and potentially isoform-specific expression patterns. Given the extensive alternative splicing of *CACNA1C*, it is also plausible that neuropsychiatric risk variants influence exon usage or isoform composition rather than total gene expression, although this possibility remains insufficiently characterized [20]. Collectively, these findings support a model in which common non-coding variants modulate disease susceptibility through subtle regulatory effects on *CACNA1C* expression and isoform balance, in contrast to rare coding mutations that produce pronounced alterations in channel function and high disease penetrance.

### 2.2. Cacna1c Rodent Models of Neuropsychiatric Disorders

Rodent models, most often mice and rats, provide a powerful translational tool for investigating the behavioral consequences of genetic variation associated with neuropsychiatric disorders [21]. There exists a broad array of standardized paradigms used to assess phenotypes relevant to neuropsychiatric disease, spanning emotional, cognitive, and social domains. Several key characteristics can be assessed across these domains to help build a more thorough phenotypic profile. For example, anxiety and locomotor-related behaviors are commonly evaluated using open-field assessment, a light–dark box, and/or an elevated plus maze [21,22]. Depression-relevant phenotypes are assessed most often using a tail-suspension task or the well-known forced swim test [23,24]. Morris water maze and spatial learning tasks can be used to evaluate learning and memory and other cognitive traits [25,26,27]. Emotional processing and associative learning are investigated using fear conditioning paradigms, in which freezing behavior, and more recently 22 kHz ultrasonic vocalizations (USVs)—thought to be indices of negative affect—are the primary readouts [28,29]. In addition, assays for social interaction and socio-affective communication, in the form of the three-chamber social approach task or direct reciprocal social interaction during social play, together with the assessment of concomitant emission of USVs, have become increasingly important for exhibiting phenotypes relevant to autism spectrum disorder and other neuropsychiatric conditions with marked social behavior and communication deficits [30,31,32,33].

Accordingly, *Cacna1c* rodent models have been examined across many of these domains and exhibit phenotypes encompassing alterations in anxiety, emotional learning, social behavior, socio-affective communication through USVs, and cognitive function.

### 2.3. In Vivo Behavioral Findings in Cacna1c Mouse Models

Several of the results obtained in mouse models for *Cacna1c* have corroborated findings seen in human *CACNA1C* data, linking anxiety and anti-depressive behavioral endophenotypes associated with neuropsychiatric disorders [34,35]. Translational relevance provided by mouse models provides insight into the role of *Cacna1c* in the neurocircuitry of neuropsychiatric disorders.

Initial studies in *Cacna1c* mouse models, commonly generated on C57BL/6 backgrounds, have established the central role of Ca_V_1.2 in the regulation of affective, cognitive, and social behaviors, with evidence implicating prefrontal cortex–amygdala–hippocampal circuitry [36]. Constitutive and region-specific reductions and deletions in *Cacna1c* expression levels in adult males and females consistently produce anxiety-like phenotypes [34,37,38,39,40]. In contrast, in a recent transgenic *Cacna1c* mouse model with increased expression levels of Ca_V_1.2, the adult mice exhibited increased risk taking and higher exploratory levels of behavior [41]. Findings related to depression-like behaviors have been less consistent, with several studies reporting antidepressant-like responses in the forced swim and tail suspension tests [34,40,42], while the overexpression of Ca_V_1.2 resulted in no depression-like behavior in the Porsolt forced swim test [41]. Reduced *Cacna1c* expression appears to strongly attenuate psychostimulant-induced locomotion in adult females but is also evident in males, indicating the role of Ca_V_1.2 in mesolimbic dopamine-dependent behaviors [34,43]. Moreover, the mouse model for Ca_V_1.2 gain-of-function characterizing TS has displayed characteristic ASD-like traits analogous to those seen to be at the core of ASD, including social and learning impairments, repetitive behavior patterns and altered vocalizations [37,44,45,46]. In contrast, the pan-neuronal overexpression of Ca_V_1.2 resulted in no deficits in social behavior in adult mice with both sexes able to distinguish a mouse from an object and intact abilities to distinguish a familiar mouse [41].

Evidence from fear conditioning paradigms has been variable, although altered fear processing, aversive learning and emotional memory processing have been reported following both loss- and gain-of-function manipulations. While some studies report minimal or no impairment, TS, characterized by gain-of-function models in adult male mice, shows enhanced contextual and cue-associated fear memory persistence [37], supporting the role of Ca_V_1.2 in aversive learning and emotional memory encoding relevant to stress- and anxiety-related pathologies. However, in the transgenic *Cacna1c* overexpression mouse model [41], the authors reported a failure in long-term memory consolidation in the adult mutant mice. Although TS knock-in mice suggest that gain-of-function Ca_V_1.2 mutations can produce distinct behavioral phenotypes relative to loss-of-function models, broader interpretations of *CACNA1C* function are complicated by heterogeneous findings across studies, including variability in behavioral directionality and molecular expression patterns [44].

*Cacna1c* has also been implicated in hippocampal-dependent cognition, with forebrain- and hippocampus-dependent deletions producing impairments in adult mice in terms of spatial learning and long-term memory, particularly under conditions of increased demand [47,48]. In contrast, adult male TS knock-in mice show relatively intact acquisition but impaired cognitive flexibility, suggesting that gain- versus loss-of-function manipulations differentially affect distinct aspects of learning and executive control [37]. More recently, integrated behavioral analysis has further refined these observations. In a cell-type- and circuit-specific *Cacna1c* deletion model targeting dopamine D1 receptor-expressing neurons, dissociable effects on emotional and cognitive domains were found, with enhanced or prolonged contextual or cued fear memory, alongside selective impairments in spatial memory performance that were dependent on sex-gene dosage [49]. Female mice showed enhanced aversive memory with partial *Cacna1c* loss, while males required a complete loss of *Cacna1c* expression to show enhanced fear behaviors [49]. For males, impairment in spatial learning was found after both partial and complete *Cacna1c* expression loss, while no effect was observed in females [49]. These findings extend previous work by highlighting that both the affected neuronal populations and biological sex critically shape *Cacna1c*-related behavioral phenotypes. Alterations in the Ca_V_1.2 channel consistently report sex-dependent effects across domains [34,50].

Alterations in social behavior in adult mice are reported, although findings remain heterogeneous. Forebrain and prefrontal *Cacna1c* deletions have been associated with reduced social preferences in some studies [42,50,51], while constitutive heterozygous models show no consistent social deficits and may even exhibit enhanced sociability in certain paradigms [37]. In the recent *Cacna1c* deletion model targeting dopamine D1 receptor-expressing neurons [49], no effect on adult social behavior was observed in males or females during a three-chamber social interaction test. In contrast, adult male mice with deletion of *Cacna1c* in CaMKII-expressing cells do show impaired social preference [43], highlighting the importance of *Cacna1c* expression in specific cell types and the role of sex in determining behavioral effects. More broadly, social phenotypes such as these are often interpreted within a neuropsychiatric framework spanning disorders characterized by altered social motivation and interaction, including autism spectrum-related characteristics among broader affective and psychotic spectrum conditions. Together, mouse models have helped establish a broader behavioral profile for *Cacna1c* across affective, cognitive and social domains, albeit with variability across manipulations and tasks with most conducted in adult mice. Given their greater suitability for complex behavioral phenotyping and more complex social repertoire from an early developmental age, rat models have increasingly been used to further probe and refine these *Cacna1c*-associated phenotypes.

### 2.4. In Vivo Behavioral Findings in the Cacna1c Haploinsufficient Rat Model

Comprehensive behavioral studies on the *Cacna1c* haploinsufficient (*Cacna1c*^+/−^) rat model have been conducted to investigate its effects on social, emotional, and cognitive behaviors over time. Similar to the mouse, behavioral measures in the *Cacna1c* rat model align with core symptoms of human neuropsychiatric disorders, such as those linked to *CACNA1C* by GWAS [52]. In a series of studies, a comprehensive gene x environmental approach was applied to assess several behavioral domains across the lifespan in a genetic rat model haploinsufficient for the cross-disorder risk gene *Cacna1c* on a Sprague–Dawley background. Of prominent note, alterations in socio-affective behavior and communication appear to be key traits in the *Cacna1c* rodent model during early life [31,53,54,55] and adulthood [56,57]. Evidence for the role of *Cacna1c* haploinsufficiency has also been shown through several studies focusing on cognitive and emotional processing [58,59,60,61].

### 2.5. Social Behavior and Communication

**Figure 2:**
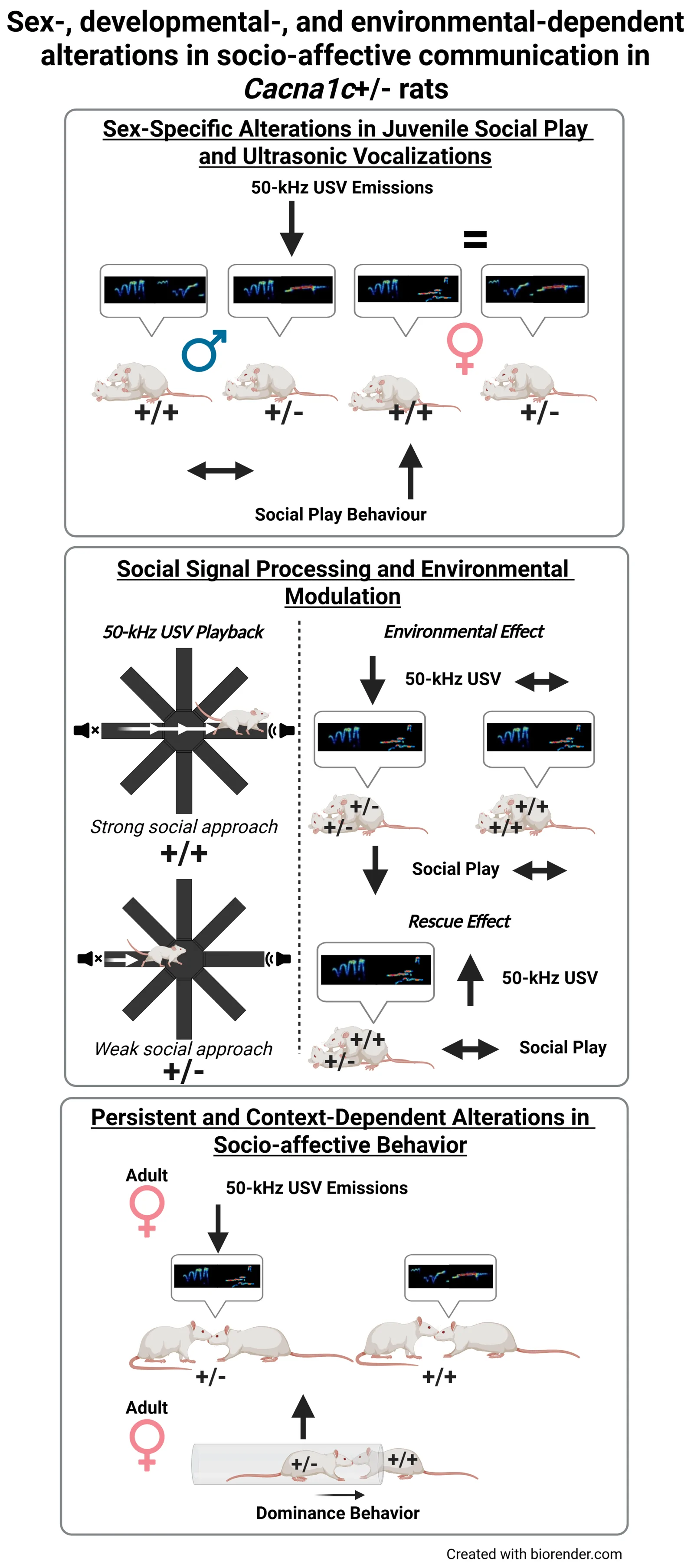
Schematic summary of sex-, developmental-, and environmental-dependent alterations in socio-affective communication observed in *Cacna1c*^+/−^ rats. The (**top panel**) illustrates the sex-specific juvenile phenotype, in which male *Cacna1c*^+/−^ rats exhibit reduced ultrasonic vocalizations (USVs) emission despite normal levels of social play, whereas female *Cacna1c*^+/−^ rats display increased social play together with altered 50 kHz USV subtype profiles. The (**middle panel**) summarizes alterations in social signal processing and environmental modulation, including reduced social approach to 50 kHz USV playback and the influence of the social environment on female social play and 50 kHz USV emission, with restoration of these behaviors following interaction with wildtype playmates. The (**bottom panel**) highlights the persistence of these phenotypes into adulthood, where female *Cacna1c*^+/−^ rats show reduced 50 kHz USV emission during social interactions together with increased social dominance behavior. Symbols indicate changes relative to wildtype controls (↑ increase, ↓ decrease, ↔ no change, = equivalent). Created in BioRender. Woehr, M. (2026) https://BioRender.com/1363hgp.

Notable social and communication alterations have been repeatedly observed in *Cacna1c*^+/−^ rats (Figure 2). These occur in both the sender and receiver as both adults and juveniles. Evidence from several studies have shown that *Cacna1c* haploinsufficiency leads to deficits in socio-affective communication, affecting both 22 kHz and 50 kHz USVs (for review see: [62]). Juvenile and adult rats produce two types of USV [63,64]. The 22 kHz “alarm calls”, usually evoked by aversive stimuli or exposure to predators, typically lead to behavioral inhibition [65,66], and 50 kHz “rat laughter” calls are associated with positive affect and rewarding experiences such as social play and mating [67,68,69]. The 50 kHz USVs are characterized by a high level of complexity and contain a variety of subtypes ranging from two to fourteen [70,71,72]. The emission and response to these prominent socio-affective stimuli appear to be altered as a result of the alteration in Ca_V_1.2 channel expression.

In juvenile rats, a highly prominent feature of their social repertoire involves social play behavior [73]. When examining social play and concomitant 50 kHz USV emission, sex-specific effects are evident. Male *Cacna1c*^+/−^ rats show typical social play behaviors but emit far fewer 50 kHz USVs compared to wildtype controls, whereas pairs of juvenile *Cacna1c*^+/−^ female rats exhibited masculinized social play—meaning a higher rate of play than what is typically observed in female rats—but had comparable 50 kHz USV emission rates to the female wildtype pairs [31,53]. While not emitting a higher number of 50 kHz calls, further analysis revealed that pairs of *Cacna1c*^+/−^ females had a distinct 50 kHz USV subtype emission pattern resembling that of male wildtype controls [54]. Upon further investigation, it was determined that environmental factors in addition to genotype strongly influenced these outcomes for juvenile females. Particularly, it was found that in juvenile *Cacna1c*^+/−^ females raised without wildtype cage mates (i.e., only with same genotype cage mates) there was a stark reduction in social play and emission of 50 kHz USVs compared to the pairs of wildtype females from same genotype cages. Interestingly, this reduction in play and 50 kHz USVs in *Cacna1c*^+/−^ females is restored by providing the opportunity to engage and play with a wildtype play partner, demonstrating a prominent rescue effect for wildtype cage mates and playmates [55].

As adults, female *Cacna1c*^+/−^ rats displayed mild social differences, showing more dominant behavior in tube tests, consistent with masculinized juvenile play behavior, but without increased aggression [56]. Adult females likewise showed a general reduction in 50 kHz USV emission during social interactions [56], suggesting that *Cacna1c* haploinsufficiency produces developmental alterations in social communication and socio-affective behaviors that are sex-specific and highly sensitive to environmental influences.

Complementing effects found in the sender, meaning the rats emitting USVs, *Cacna1c*^+/−^ rats correspondingly showed changes as receivers of social signals. To assess communicative functions of USVs, a playback paradigm can be utilized [74]. Through this technique, strong social approach behavior is elicited in response to the playback of natural 50 kHz USVs, thought to be an indication of social preference, motivation and sociability, while the presentation of natural 22 kHz USVs typically leads to behavioral inhibition, which is in line with alarm and danger signals [74]. In *Cacna1c*^+/−^ juvenile rats, social approach behavior triggered by the presentation of 50 kHz USVs was reduced in both the male and female *Cacna1c*^+/−^ rats; however, effects were more pronounced in males [53]. Moreover, after USV playback ended the wildtype controls continued to display searching behavior whereas the *Cacna1c*^+/−^ rats did not, suggesting a diminished social motivation. In line with these findings, Wöhr et al. (2020) [57] recently showed that *Cacna1c*^+/−^ rats display alterations in emotional processing in response to aversive communication signals. In *Cacna1c*^+/−^ males, but not *Cacna1c*^+/−^ females, there was a reduction in behavioral inhibition to the presentation of 22 kHz USV playback while being exposed to predator urine. This suggests that *Cacna1c* haploinsufficiency may result in lower motivation or possibly diminished capabilities to respond appropriately to socio-affective communication signals [57].

Taken together these findings indicate that *Cacna1c* haploinsufficiency affects both the production and perception of socio-affective signals, with sex and environmental factors further shaping these phenotypes. The findings from the studies outlined above are highly relevant to neuropsychiatric disorders characterized by disturbance in social functioning, motivation and emotional regulation.

Beyond the alterations in social communication and social behavior, accumulating evidence suggests that changes extend into broader domains of emotional processing and reward-related behaviors, with more subtle effects observed on cognition and learning.

### 2.6. Emotional Processing, Reward Learning and Cognition

Consistent with the notion that alterations in socio-affective signaling extend beyond social interactions alone, evidence from emotional and cognitive paradigms suggests a more subtle and heterogeneous behavioral profile in *Cacna1c*^+/−^ rats. Increased anxiety behaviors have been reported by Moon et al. (2025) [75] in adult male and female rats with a global reduction in Ca_V_1.2 expression. In addition, heightened fear responses in fear conditioning paradigms have been reported in adult *Cacna1c*^+/−^ rats relative to wildtype controls [76,77], supporting the role of Ca_V_1.2 in emotional learning and stress-related processing. Similarly, the aforementioned study showing that male *Cacna1c*^+/−^ rats displayed reduced behavioral inhibition in response to 22 kHz USV playback during predator odor exposure, further indicates altered processing of aversive socio-affective signals.

In line with findings in mice, alterations in reward-related behaviors have been observed in haploinsufficient *Cacna1c* rat models. During sign- and goal-tracking paradigms, female *Cacna1c*^+/−^ rats emitted fewer than 50 kHz USVs and displayed a shift toward goal-tracking behavior, suggesting changes in incentive salience and motivational processes [61]. In the cognitive domain, male *Cacna1c*^+/−^ rats exhibited intact spatial learning but mild impairments in cognitive flexibility during reversal learning, deficits that were ameliorated by environmental enrichment [58,59]. Similar impairments in reversal learning have been reported by Sykes et al. (2019) [76] and Moon et al. (2020) [77]. By contrast, post-weaning social isolation affected novel object recognition irrespective of genotype, whereas hippocampal overexpression of microRNA-499-5p abolished novel object preference in male *Cacna1c*^+/−^ rats, suggesting that microRNAs may modulate neuroplasticity and behavioral outcomes [60,78,79]. Finally, although Gasalla et al. (2023) [80] observed no differences in Pavlovian extinction learning, distinct regional brain activation patterns were detected in adult male *Cacna1c*^+/−^ rats, implying altered neural processing despite preserved behavioral performance.

### 2.7. Summary of Cacna1c Rodent Models

In summary, *Cacna1c* haploinsufficiency in rats appears to preferentially affect socio-affective communication and emotional processing, with comparatively subtle and task-dependent effects on cognition. Collectively, these findings suggest the role of Ca_V_1.2 in the integration of social, motivational, and affective information, indicating that disrupted socio-affective signal processing may represent a particular sensitive behavioral consequence of reduced *Cacna1c* expression.

Despite these insights, several limitations should be considered when interpreting the findings form *Cacna1c* rodent models. Interspecies differences in brain development, behavioral repertoire, and *Cacna1c* regulation limit direct extrapolation to humans and may contribute to differences between mouse and rat models. Interpretation is further complicated by the type of genetic manipulation employed, as global or constitutive reductions in *Cacna1c* expression affect multiple cell types and circuits throughout development, whereas region- and cell-specific models can reveal more selective effects. Behavioral assays likewise provide measures of specific behavioral processes rather than direct equivalents of human psychiatric symptoms and have limited predictive value in isolation. Finally, the magnitude of manipulation in haploinsufficient, knockout, or gain-of-function models differs from common human *CACNA1C* variants identified through GWAS, which typically exert modest effects within a broader polygenic and environmental context. The rodent models, therefore, should primarily be considered mechanistic tools for identifying biological and behavioral processes sensitive to altered Ca_V_1.2 signaling rather than direct representations of human *CACNA1C*-associated neuropsychiatric risk.

## 3. Cellular Mechanisms of *CACNA1C*/Ca_V_1.2 Dysfunction in the Nervous System

### 3.1. Subcellular Localization and Functions of Ca_V_1.2 in Neurons

**Figure 3:**
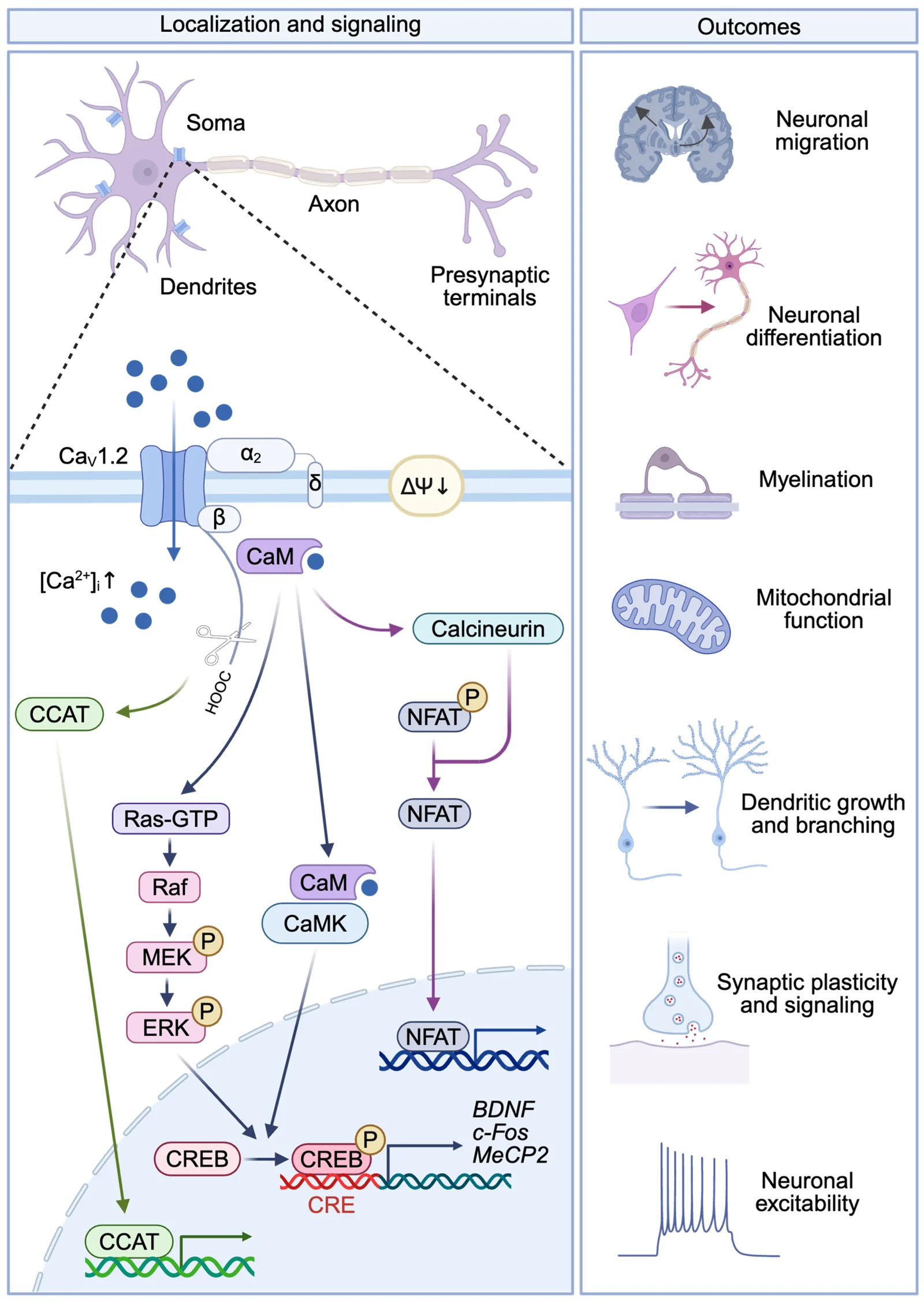
Subcellular localization, intracellular signaling and functional roles of Ca_V_1.2 in neurons. Cav1.2 channels are located primarily in the postsynaptic dendritic processes and somata and couple calcium influx to neuronal gene transcription via several intracellular signaling cascades (left side). This places Cav1.2 at a key position to regulate and influence various neuronal functions and outcomes (right side). For further details see text. BDNF, brain-derived neurotrophic factor; [Ca^2+^]_i_, intracellular calcium concentration; CaM, calmodulin; CaMK, CaM-dependent protein kinase; CCAT, calcium channel-associated transcription regulator; CREB, cyclic adenosine monophosphate (cAMP) response element-binding protein; ΔΨ, membrane potential; ERK, extracellular signal-regulated kinase; MeCP2, methyl-CpG-binding protein 2; MEK, MAPK/ERK; NFAT, nuclear factor of activated T cells; P, phosphorylated form. Created in BioRender. Königstein, D. (2026) https://BioRender.com/389grih.

Ca_V_1.2 represents the predominant L-type calcium channel (LTCC) subtype in the mammalian brain, accounting for approximately 80% of neuronal LTCCs [81,82]. Within neurons, Ca_V_1.2 is primarily localized in somato-dendritic and postsynaptic regions, where it occupies a strategic position for coupling electrical activity to intracellular calcium signals that subsequently regulate transcriptional, structural, and synaptic processes (Figure 3) [83,84]. Consequently, Ca_V_1.2 functions as a critical mediator of excitation–transcription coupling, linking neuronal activity to long-term cellular adaptations and biological processes increasingly implicated in the pathophysiology of neuropsychiatric disorders.

### 3.2. Ca_V_1.2-Mediated Intracellular Signaling and Gene Regulation in Neurons

One of the most extensively characterized functions of Ca_V_1.2 is the initiation of signaling cascades that convert transient electrical activity into sustained changes in gene expression (Figure 3). Following membrane depolarization, calcium entering through Ca_V_1.2 binds to channel-associated calmodulin (CaM), triggering the activation of multiple intracellular signaling pathways. Among the best-established pathways are the Ca^2+^/CaM-dependent protein kinase (CaMK) cascade and the Ras/mitogen-activated protein kinase (MAPK)/extracellular signal-regulated kinase (ERK) pathway [85]. The activation of these signaling networks culminates in phosphorylation of the transcription factor cyclic adenosine monophosphate (cAMP) response element-binding protein (CREB), a master regulator of activity-dependent gene expression involved in neuronal plasticity, learning, and memory. CREB-dependent transcription constitutes one of the most robust downstream consequences of LTCC activation. Genes regulated through this pathway include brain-derived neurotrophic factor (*BDNF*), *c-Fos*, methyl-CpG-binding protein 2 (*MeCP2*), and numerous plasticity-related transcripts that influence neuronal maturation and synaptic remodeling [85,86,87]. Notably, the autism-associated Ca_V_1.2^G406R^ mutant exhibits constitutive CREB activation and spontaneous transcription of activity-dependent genes (*c-Fos*, *MeCP2*), even in the absence of depolarizing stimuli, suggesting that excessive Ca_V_1.2 signaling can disrupt transcriptional homeostasis [88].

In addition to kinase-mediated pathways, Ca_V_1.2 activates calcium-dependent phosphatase signaling. Ca^2+^/CaM-dependent activation of calcineurin promotes dephosphorylation and nuclear translocation of nuclear factor of activated T cells (*NFAT*) transcription factors, thereby regulating dendritic growth, synaptic plasticity and memory formation [89]. Furthermore, the proteolytic processing of the channel can generate a C-terminal fragment known as CCAT (calcium channel-associated transcription regulator), which translocates to the nucleus and directly regulates genes involved in neuronal excitability and signaling [90].

### 3.3. Role of Ca_V_1.2 in Neuronal Migration and Differentiation

Calcium signaling regulates numerous developmental processes, including neuronal migration and differentiation, i.e., through cytoskeletal activity, making Ca_V_1.2 an important determinant of cortical organization. Evidence from TS models indicates that excessive Ca_V_1.2 activity disrupts migration of excitatory and inhibitory neurons. Prenatal expression of the TS mutation Ca_V_1.2^G406R^ in mice impairs radial migration of layer 2/3 excitatory neurons, whereas postnatal normalization of calcium influx can partially rescue these defects [91]. Human-induced pluripotent stem cell (iPSC)-derived forebrain assembloids have additionally demonstrated defects in tangential migration of TS inhibitory interneurons, characterized by shorter saltation length and increased saltation frequency that can be corrected by LTCC inhibition with nimodipine [92].

Ca_V_1.2 also influences the differentiation of neural progenitors. Studies using neuronal cultures differentiated from TS patient-derived iPSCs and mice overexpressing Ca_V_1.2^G406R^ in neurons have demonstrated reduced numbers of lower-layer cells expressing SATB2, thereby decreasing the fraction of callosal projection neurons [93,94]. Conversely, *Cacna1c* knock-out increased SATB2 expression in the developing cortex of mice, indicating that calcium influx through Ca_V_1.2 contributes to developmental programs controlling neuronal identity [93].

### 3.4. Regulation of Neuronal Morphology, Synaptic Plasticity, and Circuit Function by Ca_V_1.2

Ca_V_1.2-mediated calcium signaling exerts profound effects on neuronal morphology and connectivity. In both rodent and human TS models, mutant channels cause activity-dependent dendritic retraction and reduce the number of dendritic branches. These structural abnormalities are independent of calcium flux through TS-Ca_V_1.2 and associated with activation of GTPase RhoA signaling that governs cytoskeletal remodeling [95]. In addition to dendritic development, Ca_V_1.2 regulates axonal growth and connectivity. Expression of gain-of-function Ca_V_1.2 channels in cortical layer 2/3 excitatory neurons diminished axonal arborization in the contralateral hemisphere [96]. Furthermore, enhanced maturation of oligodendrocyte progenitor cells isolated from cortex and increased axon myelination in the corpus callosum have been reported for TS mice [97].

At the synaptic level, Ca_V_1.2 contributes to neuronal excitability and activity-dependent plasticity. Forebrain-specific deletion of *Cacna1c* leads to increased presynaptic excitatory inputs as well as higher frequency and amplitude of miniature excitatory postsynaptic currents in layer 5 pyramidal neurons, suggesting a shift in the excitation-inhibition balance, a circuit feature frequently implicated in neuropsychiatric disorders [42]. Moreover, Ca_V_1.2 is required for specific forms of hippocampal long-term potentiation. Loss of Ca_V_1.2 function impairs MAPK/CREB-mediated transcription in CA1 pyramidal neurons, linking channel activity to molecular mechanisms underlying spatial memory formation [47].

### 3.5. Impact of Ca_V_1.2 on Mitochondrial Function, Oxidative Stress, and Neuronal Survival

Recent studies have extended the role of Ca_V_1.2 beyond neuronal signaling and development to the regulation of mitochondrial homeostasis and cellular resilience. In immortalized mouse hippocampal neurons, both pharmacological LTCC inhibition and siRNA-mediated reduction in *Cacna1c* expression protect against glutamate-induced oxidative damage, preserving mitochondrial membrane potential, maintaining ATP levels, and preventing cell death [98,99]. These findings suggest that Ca_V_1.2 occupies a critical position at the intersection of neuronal activity, energy metabolism, and stress responses. Dysregulated calcium entry may therefore contribute not only to developmental abnormalities and synaptic dysfunction but also to cellular vulnerability under pathological conditions. However, since this is a developing field of research, further validation of these results is still needed.

### 3.6. Potential Role of Ca_V_1.2 Signaling in Neuroinflammatory and Peripheral Immune Signatures

In contrast to excitable cells such as neurons, cardiac and smooth muscle, where LTCCs are primarily activated by membrane depolarization, the mechanisms governing LTCC activity in non-excitable cells such as microglia and peripheral immune cells have not yet been fully elucidated and therefore represent a still evolving research field [100,101].

In microglia, the brain’s resident immune cells, intracellular calcium dynamics regulate phagocytosis, oxidative burst, inflammasome activation, and subsequent cytokine release. These signals arise from a network of purinergic receptors, store-operated calcium entry, transient receptor potential channels, intracellular stores, and ryanodine receptors (RyRs) [102,103]. LTCC-related pathways may thus contribute to microglial phenotype and function under inflammatory conditions [101]. Although Ca_V_1.2 is not regarded as the dominant calcium entry route in resting microglia, it may act as context-dependent modulator of reactive states. Several studies combining channel detection with genetic manipulation provide evidence for Ca_V_1.2 expression and activity in microglia. Espinosa-Parrilla et al. [104] showed that activated BV2 microglia express both Ca_V_1.2 and Ca_V_1.3 (and that nifedipine reduced TNF and NO release without affecting their phagocytotic capacity or viability). Ca_V_1.2 activity has further been proposed to functionally regulate intracellular calcium fluctuations that affect microglial activation, migration, and the subsequent release of pro-inflammatory cytokines [101]. Direct evidence is provided by demonstrating that pharmacological blockade or knockdown of microglial Ca_V_1.2 can disrupt microglial balance, promoting neurotoxic states, accelerating neurodegeneration, and exacerbating neuroinflammation in a murine model of Parkinson’s disease [105].

Simultaneously, findings suggest that *CACNA1C*/Ca_V_1.2 expression in astrocytes may help to maintain the structural and functional integrity of the blood–brain barrier (BBB). In brain-specific *Cacna1c*-deficient mice, the deficiency or dysfunction in astrocytic Ca_V_1.2 triggers severe astrocyte reactivity, causing upregulation of aquaporin-4 at astrocytic endfeet, destabilizing the BBB, and inducing a local increase in chemokines and inflammatory mediators [106].

In addition, the immunological influence of *CACNA1C*/Ca_V_1.2 extends beyond the CNS, driving systemic immune responses that engage in cross-talk with neural pathology. As described before, pathogenic mutations, such as the gain-of-function variants underlying TS, have been linked to peripheral immune deficiencies and systemic cytokine dysregulation alongside severe neurodevelopmental deficits [7,100]. Although it results from rare, highly penetrant variants, it illustrates how altered Ca_V_1.2 function can affect multiple organ systems, including the CNS and the peripheral immune system.

Ca_V_1.2 expression has been found in several cell types of both the innate and the adaptive immune system [100,107]. Human dendritic cells, for example, express Ca_V_1.2 functionally coupled to RyRs. In these cells, LTCC–RyR signaling has been shown to induce upregulation of CD83 and MHC class II surface molecules, evidencing the role of Ca_V_1.2 in dendritic cell maturation and antigen presentation [108]. It has further been proposed that Ca_V_1.2 (or related LTCC components) may contribute to T-cell-receptor-induced calcium signaling, Th2 differentiation, and cytokine production, since Ca_V_1 knockdown reduced Th2 activation and ameliorated experimental asthma in mice [109,110]. However, this view of the role of Ca_V_1.2 in immune cells has been challenged by findings demonstrating that voltage-gated calcium channels (Ca_V_1.1 [111]; Ca_V_β [112]) do not function as ion channels in T cells [113] and regulate cellular function independently of voltage-gated ion flux [112] through protein interactions, scaffolding or modulation of gene expression (Ca_V_1.1 [114]; Ca_V_β [115]). As the topic of Ca_V_ biology in peripheral immune cells lies beyond the scope of the present review, readers are referred to comprehensive reviews in the field [100,107,116,117,118]. Whether Ca_V_1.2 similarly mediates non-canonical signaling or scaffolding functions in immune cells remains to be established.

When astrocytic *CACNA1C*/Ca_V_1.2 dysfunction compromises the BBB, this peripheral–central divide collapses. Concomitantly, enhanced dendritic cell maturation may promote T-cell activation and release of pro-inflammatory mediators. Soluble inflammatory factors and, in severe cases of BBB disruption, also peripheral immune cells like monocytes and neutrophils, may gain access to brain parenchyma. This infiltration could amplify local neuroinflammation, reshape peripheral immune differentiation by microglia-secreted inflammatory mediators and thus create a feedback loop of chronic systemic immune activation. Consequently, *CACNA1C*/Ca_V_1.2 can be regarded as an immunomodulatory hub [100], whose dysregulation couples local glial reactivity with systemic immune dysfunction, thereby potentially modulating the onset and progression of neuropsychiatric and neurodegenerative disorders [106]. Whether *CACNA1C*/Ca_V_1.2 regulation associated with immune cell reactivity in neural disease can be exploited therapeutically needs to be further clarified in appropriate studies on disease-relevant genetic variants and targeted pharmacological interventions.

### 3.7. Summary of Ca_V_1.2 Dysfunction in Neuronal and Non-Neuronal Cells in the CNS

Ca_V_1.2 functions as a central molecular hub linking neuronal activity to intracellular signaling, gene transcription, cellular development, and circuit plasticity (Figure 3). Genetic evidence from TS and large-scale GWAS has firmly established *CACNA1C* as a major neuropsychiatric risk gene, while mechanistic studies have revealed how altered Ca_V_1.2 signaling can influence neuronal migration, differentiation, dendritic architecture, synaptic plasticity, and cell survival. Further, *CACNA1C* mutations have been linked to altered immune responses in non-excitable cells such as in microglia and astrocytes, as well as to pathology-associated peripheral immune signatures, suggesting Ca_V_1.2 is an immunomodulatory hub of neuro-immune cross-talk in neurodevelopmental and neuropsychiatric disorders. Together, these findings support a model in which perturbations of Ca_V_1.2-dependent signaling could represent a convergent mechanism underlying vulnerability to neurodevelopmental and neuropsychiatric disorders. However, important limitations remain, particularly regarding the cell-type-specific functions of Ca_V_1.2 in glia, its relationship to mitochondrial dysfunction, and the mechanisms by which altered Ca_V_1.2 signaling intersects with immune pathways. The extent to which these processes represent primary drivers, downstream consequences, or interacting components of disease pathology has yet to be fully elucidated and warrants further investigation.

## 4. *CACNA1C*/Ca_V_1.2 in the Heart

**Figure 4:**
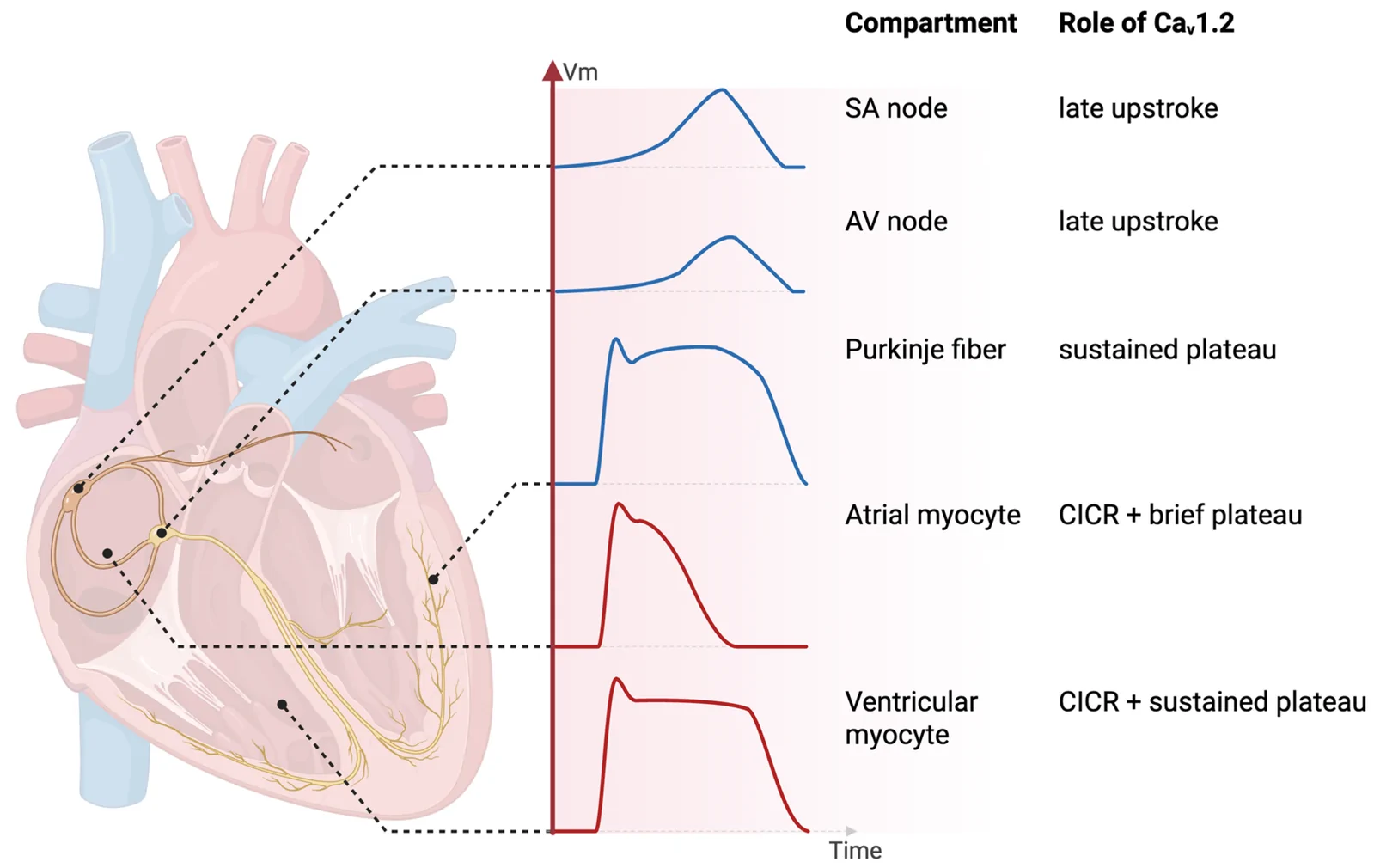
Compartment-specific AP morphologies and roles of Ca_V_1.2 in the mammalian heart. Left, schematic cardiac anatomy and conduction pathways. Middle, representative action potential shapes of nodal and conduction system cells (blue) and atrial and ventricular myocytes of the working myocardium (red); traces are schematic and not to scale. Right, principal compartment-specific contributions of Ca_V_1.2. For further details see text. Abbreviations: AV, atrioventricular; CICR, calcium-induced calcium release; SA, sinoatrial. Created in BioRender. Königstein, D. (2026) https://BioRender.com/su70kpt.

Ca_V_1.2 is expressed throughout the mammalian heart, but its roles vary by cardiac compartment [3,4] (Figure 4), shaping how *CACNA1C* variants may cause disease. By converting membrane depolarization into calcium entry, Ca_V_1.2 supports nodal impulse formation and, in working myocardium, sustains the action potential (AP) plateau and couples excitation to contraction. In the sinoatrial (SA) node, Ca_V_1.2 is recruited mainly during the later phase of the upstroke of the AP. In the atrioventricular (AV) node, Ca_V_1.2 contributes to the slow upstroke of the AP. Thus, LTCC blockers like verapamil impair SA and AV nodal APs and exert negative-chronotropic and negative-dromotropic effects. These effects have led to the use of cardiac-specific LTCC blockers (verapamil, diltiazem) as anti-arrhythmic drugs (AADs) [119,120], e.g., in patients with atrial fibrillation (AF). Cardiac Purkinje myocytes exhibit a sustained plateau phase (similar to ventricular myocytes), and this plateau phase is largely mediated by Ca_V_1.2 [121]. In atrial and ventricular myocytes, Ca_V_1.2 triggers calcium-induced calcium release (CICR) and, hence, is essential for cardiac excitation–contraction coupling. Interference with this process, e.g., by LTCC blockers, results in impaired cardiac contractility (negative-inotropic effect), an effect that prohibits the use of cardiac-specific LTCC blockers (class IV AADs) in patients with heart failure (HF) (with reduced ejection fraction) [119,122].

In summary, Ca_V_1.2 exerts various pivotal functions in both pacemaker cells and working cardiomyocytes, which makes the heart particularly vulnerable to alterations in Ca_V_1.2 expression and function.

The cardiac phenotype of *CACNA1C* dysfunction is variant- and mechanism-dependent rather than uniform. Gain-of-function variants underlying TS and isolated long QT syndrome type 8 (LQT8) constitute the strongest gene–disease evidence for *CACNA1C* in cardiac disease. By contrast, loss-of-function alleles and the proposed *CACNA1C*–Brugada syndrome association remain mechanistically plausible but weakly supported at the gene level; each reported variant therefore needs to be assessed individually, using clinical presentation, family segregation and functional data. Across these mechanistic axes, regulatory layers that dynamically tune Ca_V_1.2 function—for example the β-adrenergic signaling node—help explain why reduced channel expression does not necessarily mean reduced basal current density [6,123,124] and why biophysically identical variants can produce different clinical phenotypes (TS1 vs. TS2) [125].

### 4.1. Gain-of-Function Variants: Timothy Syndrome and Long QT Syndrome 8

#### 4.1.1. Timothy Syndrome (TS): A Splice-Variant-Graded Severity Spectrum

The best-characterized cardiac manifestation of multisystem *CACNA1C* dysfunction is TS [7]. The canonical TS1 mutation, G406R in the alternatively spliced exon 8A, markedly impairs voltage-dependent inactivation of Ca_V_1.2, producing a sustained inward calcium current that prolongs the cardiac AP and underlies QT prolongation and arrhythmia susceptibility. Clinically, QTc typically exceeds 500 ms, accompanied by a prolonged isoelectric ST segment, functional 2:1 AV block, T-wave alternans, and a high risk of torsade-de-pointes and ventricular fibrillation. In contrast to the predominantly cardiac gain-of-function phenotypes discussed later, TS1 is defined by multisystem involvement, including neuropsychiatric features (see above), syndactyly, immune dysregulation and intermittent hypoglycemia.

TS2 is caused by the identical G406R substitution in exon 8 rather than exon 8A and carries an even more severe cardiac phenotype [125]. The severity gradient is quantitative and follows splice-isoform abundance: the dominant cardiac Ca_V_1.2 splice variant (exon 8) comprises ≈80% of cardiac transcripts [125,126]. The minor isoform (exon 8A/α1C-b) is predominantly expressed in vascular smooth muscle and developmentally enriched in neurons. Splicing between exons 8 and 8A is regulated by polypyrimidine tract-binding protein (PTB) and its neuronal homolog nPTB [126]. A TS1 (exon 8A) mutation therefore affects a small fraction of cardiac Ca_V_1.2; a TS2 (exon 8) mutation affects the dominant pool—hence the earlier-onset and more lethal phenotype of TS2. The same inactivation defect therefore produces different severities depending on how much of cardiac Ca_V_1.2 it affects.

Like the exon-8 G406R variant, G402S markedly impairs voltage-dependent inactivation. Because exon 8-containing Ca_V_1.2 transcripts predominate in the heart (~80% of cardiac transcripts), G402S is associated with a severe cardiac TS2 phenotype with marked QT prolongation and life-threatening ventricular arrhythmias [125,127]. G419R, by contrast, represents a mechanistically distinct gain-of-function variant. Rather than impairing inactivation, G419R produces an increase in peak calcium current density and a hyperpolarizing shift in activation gating linked to calmodulin-dependent regulation, thereby enhancing Ca_V_1.2 activity at physiological membrane potentials [128,129]. Clinically, G419R has been associated with TS features together with left ventricular non-compaction and ventricular pre-excitation via multiple accessory pathways, a phenotype not previously reported in TS. In a second family, an alternative nucleotide substitution at the same codon (c.1255G>A) produced the same p.G419R amino-acid change and was associated with familial transmission and survival into middle adulthood [130]. Together, G402S and G419R illustrate that pathogenic *CACNA1C* gain of function can arise through distinct gating abnormalities and can produce a broad spectrum of clinical severity.

The recent international Delphi-consensus statement of the Timothy Syndrome Alliance [12] retains the designations TS1 (syndactyly—featuring classical exon-8A p.G406R phenotype) and TS2 (hip dysplasia—featuring exon-8 variants, including p.G406R and p.G402S as in Splawski’s original definition), while introducing “*CACNA1C*-Related Disorder (CRD)” as an umbrella term for syndromic *CACNA1C* variants that do not fulfill the classical TS constellation. Attenuated familial presentations, such as the G419R phenotype reported by Fanucci et al. (2025) [130], also fall within this expanded framework. The CRD terminology thus accommodates an increasingly broad phenotypic and genetic spectrum, including recently reported variants such as p.C1021R [131].

Borbás et al. (2022) [132] systematically reviewed 59 TS, 6 cardiac-only TS (COTS), and 20 isolated LQT8 index cases, documenting significantly earlier onset and higher mortality in TS than in either COTS or LQT8. Congenital heart defects—including patent ductus arteriosus, ventricular septal defects, and tetralogy of Fallot—recur in the case literature, although precise frequencies are difficult to establish given disease rarity and probable publication bias toward severe presentations. Delinière et al. (2023) [127] further characterized the G402S variant, and Han et al. (2019) [133] and Jiang and Zhang (2023) [134] reviewed the mechanistic and clinical spectrum from cell to bedside. Across the spectrum, impaired channel inactivation is the shared molecular lesion, whereas clinical expression is modulated by splice-isoform context, variant-specific biophysics, and likely genetic background.

#### 4.1.2. Cardiac-Only TS and Isolated LQT8

Not all gain-of-function variants produce the complete TS phenotype. Some cause severe QT prolongation, structural abnormalities, and sudden death without multisystem developmental features; the extra-cardiac TS phenotype appears to depend on mutation-specific biophysical properties and their developmental timing. Boczek et al. (2015) [135] identified R518C in a pedigree with long QT (LQT) interval, hypertrophic cardiomyopathy, congenital heart disease, and sudden cardiac death, revealing a mixed gain- and loss-of-function biophysical profile—a finding that remains mechanistically unresolved. One possibility is that the variant has differential effects on distinct gating states or in cellular contexts not fully captured by heterologous expression systems. Gakenheimer-Smith et al. (2021) [136] documented AF and sick sinus syndrome in carriers of the same variant across four generations. This finding illustrates both within-pedigree variability and the extension of *CACNA1C* dysfunction to supraventricular and conduction tissue, consistent with the established role of Ca_V_1.2 in SA pacemaker APs and atrial AP duration. Fukuyama et al. (2014) [137] identified five novel mutations in 2.5% of LQT syndrome (LQTS) probands, with phenotypes ranging from mild QT prolongation to near-TS severity. Wemhöner et al. (2015) [138] helped define non-syndromic LQT8 as a clinical entity distinct from TS. Whether the current three-tier classification (TS/COTS/LQT8) adequately captures the data, or whether a grouping based on the underlying molecular mechanism would be more informative, remains an open question.

Taken together, *CACNA1C* gain-of-function disease is not uniform and best understood by the specific biophysical defect and the developmental context in which that defect operates.

### 4.2. Loss-of-Function Variants: Brugada Syndrome Type 3 and Short QT Syndrome

Loss-of-function variants reduce L-type calcium current and produce an electrophysiologically distinct phenotype. Antzelevitch et al. (2007) [139] identified *CACNA1C* mutations (A39V, G490R) in patients with ST segment elevation, short QT intervals (≤360 ms), and sudden cardiac death, leading to the historical designation Brugada syndrome type 3 (BrS3). Subsequent gene–disease validity reappraisal has substantially weakened this association. *SCN5A* remains the only gene with definitive evidence for BrS causality, whereas *CACNA1C* and other historically implicated BrS genes are considered to have disputed gene–disease validity [140,141]. Consistent with this reassessment, the historically prominent variant G490R occurs in population databases at a frequency that is difficult to reconcile with a rare, highly penetrant arrhythmia syndrome.

The proposed mechanism is physiologically plausible: reduced L-type calcium current could increase repolarization heterogeneity and thereby favor an arrhythmogenic substrate. However, mechanistic plausibility alone does not establish gene-level causality, and current evidence does not support *CACNA1C* as an established monogenic cause of classic BrS. This contrasts with the *CACNA1C*–Timothy syndrome/LQT8 spectrum, for which gain-of-function variants are supported by substantially stronger genetic, functional and mechanistic evidence.

### 4.3. CACNA1C Haploinsufficiency and Regulatory Control of Ca_V_1.2

#### 4.3.1. Cardiac Consequences of Cacna1c Haploinsufficiency

The human cardiovascular consequences of isolated *CACNA1C* haploinsufficiency remain poorly defined, and most data derive from rodent models. As mentioned before, constitutive homozygous loss of *Cacna1c* is lethal in mice before embryonic day 14.5 [5]. Using a cardiomyocyte-specific, tamoxifen-inducible Cre approach (α-MHC-MerCreMer × Cacna1c-floxed mice, with loxP sites flanking exon 2; 6–7-week-old mice of either sex), Rosati et al. (2011) [6] showed that adult homozygous deletion of *Cacna1c* is lethal within ≈12 days, with myocardial contractility falling to ≈14% of control. This has restricted in vivo work on chronically reduced expression of Ca_V_1.2 to heterozygous (*Cacna1c*^+/−^) and tissue-restricted conditional models.

Two constitutive heterozygous models have been used extensively in this context: a global *Cacna1c*^+/−^ mouse [142] and a global *Cacna1c*^+/−^ rat carrying a zinc-finger-nuclease-generated 4 bp deletion in exon 6 that results in a premature stop codon [123,124]. In the rat studies discussed here, female animals aged 9–15 months were analyzed [123,124]. Both models are viable and do not appear to exhibit excess mortality. Their cardiac phenotype—at least under basal, non-stressed conditions—is surprisingly mild [123,142], in particular when considering the many pivotal functions of Ca_V_1.2 in the heart and the severe cardiac phenotype observed in TS (including lethal arrhythmias). The heart rate in *Cacna1c*^+/−^ mice is slightly increased. The heart weight to body weight ratio is normal in *Cacna1c*^+/−^ rats [123] or increases only at older ages in *Cacna1c*^+/−^ mice [142]. Cardiac contractility (as judged from echocardiography or sarcomere/cell shortening measurements in ventricular myocytes) is slightly decreased (mice [142]) or unchanged (rats [123]). Similarly, ventricular myocyte calcium transients and SR calcium load are only slightly decreased (mice) or essentially unchanged (rats). Finally, and in line with the contractility and calcium data, L-type calcium current is either only slightly reduced (mice) or unchanged (rats). These observations imply that—in both rodent models—the hearts and cardiomyocytes must have evolved mechanisms to compensate for the loss of one *Cacna1c* allele and retain (almost) normal function.

In both cases, these compensatory mechanisms converge on altered intracellular calcium regulation and altered kinase or phosphatase signaling to maintain cellular function, but they come at the expense of compromised responses to stress. In *Cacna1c^+/–^* mice [142], paradigms of pathological or physiological stress, i.e., pressure overload by aortic constriction, chronic isoproterenol (a β-adrenergic receptor agonist) infusion, or forced swimming, caused exaggerated hypertrophy, ventricular dysfunction, and dilation. This cardiac phenotype could be explained by elevated neurohumoral stress resulting (1) in sensitized SR calcium release with leaky type-2 ryanodine receptors (RyR2s) with augmented phosphorylation at Ser-2808 and Ser-2814 to compensate for reduced calcium influx via Ca_V_1.2 and (2) in augmented calcineurin signaling to drive the exaggerated hypertrophy response [142]. On the other hand, cardiomyocytes from *Cacna1c^+/–^* rats exhibited an impaired sympathetic stress response [123]. Here, the isoproterenol-induced increase in cytosolic calcium transients and contractility was blunted due to elevated PKA-dependent phosphorylation of RyR2 (at Ser-2808) and of Ca_V_1.2 (at Ser-1928; rat/rabbit numbering used here; Ser-1927 in mouse/guinea-pig; the corresponding distal C-terminal residue is annotated as Ser-1981 in canonical human α1C, UniProt Q13936-1, based on the sequence alignment in [143]; see also Figure 1) under baseline conditions, resulting in a compromised sympathetic reserve [123,124].

The conceptual lesson from the *Cacna1c^+/–^* mouse and rat models is that the heart exhibits remarkable plasticity in compensating for reduced *Cacna1c* expression to retain an apparently (close to) normal function under basal, non-stressed conditions [6,123,142] and that physiological and pathological stressors unveil a cardiac phenotype characterized by an inability to respond adequately to sympathetic and pressure overload stress [123,142]. Whether the rodent models generalize to the human heart remains unknown.

#### 4.3.2. Regulatory Control of Ca_V_1.2: β-Adrenergic Regulation and Beyond

The clinical phenotype of a *CACNA1C* variant depends not only on its intrinsic biophysical effect but on regulatory layers that dynamically shape channel availability, gating, and expression. Four axes are most relevant in the cardiac setting: β-adrenergic regulation, CaMKII-mediated facilitation, redox modulation, and transcriptional/post-transcriptional control. Rather than reviewing each axis in mechanistic depth, this and the following section highlight how these regulatory layers intersect with a central paradox of *Cacna1c* haploinsufficiency: loss of one *Cacna1c* allele would predict a proportional halving of Ca_V_1.2 expression and sarcolemmal L-type calcium influx, yet Ca_V_1.2/α_1C_ protein falls by only ≈20–40% and peak L-type calcium current is either essentially unchanged or reduced by only ≈25% across *Cacna1c*^+/−^ mouse and rat models [6,123,124,142].

Physiologically, sympathetic activation is arguably the most important means of augmenting cardiac contractility, and this is achieved largely by β-adrenergic stimulation of L-type calcium current and elevated cytosolic calcium transients [144]. β-adrenergic activation leads to a huge increase in L-type calcium current through PKA-dependent phosphorylation of the channel macromolecular complex. The field has shifted from an α_1C_-centered phosphorylation model (at Ser-1700/Thr-1704 and Ser-1928/1981, Figure 1) toward a Rad-centered model in which PKA phosphorylation of the RGK protein Rad releases its inhibitory grip on channel gating [145,146,147,148], mechanistically analogous to PKA phosphorylation of phospholamban relieving the inhibition of SERCA during β-adrenergic stimulation. In 4SA-Rad knock-in mice, prevention of Rad phosphorylation markedly attenuates β-adrenergic augmentation of Ca^2+^ influx and contractility [147,149,150]. The additional contribution of direct α_1C_ phosphorylation, however, particularly at Ser-1700/Thr-1704, remains an option [151,152]. In conclusion, PKA-dependent phosphorylation of Rad is now widely accepted as the primary mediator of L-type calcium current elevation during β-adrenergic stimulation in cardiomyocytes. Direct α_1C_ phosphorylation by PKA, on the other hand, may serve a secondary modulatory role in L-type calcium current regulation.

Beyond β-adrenergic regulation, Ca_V_1.2 activity is further shaped by additional means including CaMKII-dependent facilitation and redox modulation acting through α_1C_ thiol oxidation and CaMKII activation, which becomes pathologic in heart failure [153,154,155]. Total channel abundance is further set by transcriptional and post-transcriptional inputs, notably mineralocorticoid-driven *Cacna1c* promoter switching [156] and miR-328/miR-499/miR-155 activity in AF [157,158,159]. It remains unknown though whether any of these axes is operating in hearts of animals with *Cacna1c* haploinsufficiency.

#### 4.3.3. The Paradox of Preserved Calcium Current in Cacna1c Haploinsufficiency

A central mechanistic puzzle in *Cacna1c* haploinsufficiency is that loss of one gene copy does not produce a proportional loss of Ca_V_1.2 expression and L-type calcium current. Multiple independent studies on mouse and rat models converge on a mismatch across the gene-to-current pathway in adult ventricular myocytes: In a mouse model, mRNA was reduced to ≈60% and Ca_V_1.2 protein to ≈80% of control, while L-type current was not significantly reduced [6]. In another mouse model, Ca_V_1.2 protein was reduced to ≈60% of control, i.e., by ≈40%, yet L-type current was reduced by only ≈25% [142]. Finally, in a *Cacna1c*^+/−^ rat model, Ca_V_1.2 protein was reduced to ≈70% of control, but neither L-type current nor sarcolemmal calcium influx were changed [123,124].

Several cellular mechanisms could contribute to this compensation, and a further observation frames the altered local regulatory context in which they operate:

(i) Post-transcriptional or proteostatic buffering: Rosati et al. (2011) [6] proposed that the mismatch reflects a passive bottleneck: one of the biosynthetic steps downstream of transcription becomes saturated—for example, ER folding capacity or the pool of β and α_2_δ auxiliary subunits—so that halving mRNA does not translate into halving protein. Faster translation or slower protein degradation could also contribute, but neither has been demonstrated in *Cacna1c*^+/−^ hearts.

(ii) Preferential preservation of the functional surface pool. Reduced total Ca_V_1.2 protein need not translate into a proportional reduction in sarcolemmal, dyadically localized channels: preferential membrane retention, altered turnover of surface-resident channels, increased clustering, altered trafficking or shifts in auxiliary subunit stoichiometry could each preserve the gating-competent channel count in the membrane [160,161]. Königstein et al. (2024) [124] found unchanged total Ca_V_β_2_ protein in *Cacna1c*^+/−^ rat ventricles. This means that—because total α_1C_ expression is reduced—the bulk Ca_V_β_2_-to-α_1C_ abundance ratio is increased. Whether this alters subunit availability, channel assembly, or surface delivery remains unknown. The general possibility of a preserved functional surface pool remains untested and would require quantitative surface protein/channel measurements.

(iii) Altered local PKA-mediated phosphorylation of Cav1.2 and Rad in the vicinity of the channel: As mentioned above, Rad is an important regulator of channel activity. Rad holds a fraction of surface Ca_V_1.2 channels in a low open probability state, and PKA phosphorylation of Rad releases this brake. Thus, increased Rad phosphorylation would recruit these “reserve” channels. In the *Cacna1c*^+/−^ mouse, reduced L-type current and contractile performance were accompanied by mild tachycardia, and metoprolol (a β-adrenergic receptor blocker) attenuated the disease phenotype, consistent with compensatory neuroendocrine activation [142]. Such β-adrenergic drive could, in principle, increase tonic Rad phosphorylation and recruit reserve channels; however, Rad phosphorylation and its contribution to basal current have not been examined directly in *Cacna1c*^+/−^ myocardium.

Along these lines, basal PKA phosphorylation of α_1C_ at Ser-1928 was found to be increased in *Cacna1c*^+/−^ rat ventricles [124]. While phosphorylation of this site might not be required for β-adrenergic augmentation of the L-type calcium current in cardiomyocytes [162], it nevertheless serves as a sensitive readout for local PKA activity in the vicinity of the channel and hence suggests that, under basal conditions, Ca_V_1.2-mediated calcium influx is normalized in the face of reduced channel number, possibly by elevated PKA-dependent phosphorylation of the Ca_V_1.2 macromolecular complex, including Rad.

Taken together, these findings converge on a consistent phenotype in cardiomyocytes: basal Ca_V_1.2 function, i.e., L-type calcium current, is largely preserved despite reduced *Cacna1c* expression due to an elevated basal PKA-mediated phosphorylation of the channel and presumably Rad; this basal localized PKA “hyperphosphorylation” leaves a less sympathetic reserve for channel regulation. Hence, during acute sympathetic stress, the cardiomyocytes are unable to recruit a sufficient number of additional channels—due to an already exhausted PKA phosphorylation reserve—resulting in impaired contractile function. The phenotypic manifestations of this exhausted reserve are consistent across models: exaggerated hypertrophy, ventricular dysfunction and dilation after pressure overload, chronic isoproterenol infusion or forced swimming in the *Cacna1c*^+/−^ mouse [142], and blunted inotropic and Ca^2+^-transient responses to isoproterenol in the *Cacna1c*^+/−^ rat, despite preserved basal function [123,124]. The concept of an exhausted sympathetic reserve thus provides a mechanistic framework linking preserved basal function to altered cardiac responses under stress in *Cacna1c* haploinsufficiency.

## 5. Clinical Perspective: Killing Two Birds with One Stone by Targeting *CACNA1C*/Ca_V_1.2?

In clinical practice, there are mutual associations between neuropsychiatric disorders, like major depression, bipolar disorder, autism or schizophrenia, and many cardiovascular diseases including hypertension, coronary artery disease (CAD), HF or AF. For example, depression may increase the risk for CAD, myocardial infarction, HF or AF; vice versa, patients with AF or CAD have an increased risk of developing depression [163,164,165,166,167,168]. Patients with schizophrenia have reduced life expectancy (by 10–20 years); this is caused, in large part, by increased cardiac mortality, with mortality rates for CAD, myocardial infarction, HF, and cardiac arrhythmias being increased by a factor of about 2–3 each [169,170]. Likewise, genetic evidence strongly implicates *CACNA1C* variants in many neuropsychiatric disorders as well as cardiovascular disease (as already discussed above). Data from rodent models largely confirm that altered expression and function of Ca_V_1.2 may affect both neurological function and behavior as well as cardiac function. This suggests that Ca_V_1.2 may be a common mechanistic link between neuropsychiatric and cardiac disorders and that appropriate targeting of Ca_V_1.2 may be used to treat both neuropsychiatric and cardiac disease in a given (selected) patient at the same time.

LTCC blockers (CCBs) have been used clinically for decades to treat cardiac arrhythmias (as class IV AADs like verapamil and diltiazem), CAD (as anti-anginal drugs, DHPs and non-DHPs) and hypertension (as anti-hypertensive drugs, DHPs and non-DHPs). Thus, there is ample clinical evidence regarding their desired effects but also their unwanted side effects, and both can be explained well by their inhibition of LTCCs in the respective tissues and cell types. These include (1) negative chronotropic, dromotropic and inotropic effects in the heart due to inhibition of LTCCs in SA and AV nodal pacemaker cells and ventricular myocytes; (2) the dilation of coronary arteries due to inhibition of LTCCs in coronary artery smooth muscle cells; and (3) reduction in blood pressure due to inhibition of LTCCs in arterial smooth muscle cells of the peripheral circulation. The (4) latter effect on arterial smooth muscle cells may also facilitate the development of flush and ankle edema, frequently observed side effects of CCBs. Finally, they include (5) obstipation caused by impaired intestinal motility due to inhibition of LTCCs in intestinal smooth muscle cells. Whether CCBs also act in the brain to inhibit LTCCs in neurons and non-neuronal cells and whether this might be exploited clinically to treat neurological or neuropsychiatric disorders is less clear though. Clinical studies in the psychiatric setting conducted so far often suffer from limitations such as small sample size, potential indication bias, or being retrospective and observational and the overall evidence in support of the clinical utility of CCBs in such settings remains controversial with some studies hinting at a potential benefit while others do not confirm any such effect or even report adverse outcomes. For example, six double-blind trials found no evidence for an effect of verapamil in acute mania [171]. However, RCTs were small (157 patients in total) and of short duration. In the four trials in which verapamil was compared to lithium, lithium treatment was found not to be superior to verapamil [171]. In a Mendelian randomization study, genetically proxied CCBs showed no protective effect on mental disorders [172]. Some large observational studies, however, reveal more promising results. In a study with >140,000 individuals from the Swedish population with bipolar disorder, schizophrenia or nonaffective psychosis, use of CCBs was associated with reduced rates of psychiatric hospitalization and self-harm in all three subgroups [173]. In two studies with individuals from the Finnish population with either bipolar disorder (>60,000) or schizophrenia (>61,000), the use of CCBs was associated with a reduced risk of hospitalization due to affective symptoms [174] or a reduced risk of psychiatric rehospitalization [175], respectively. Interestingly, in both studies, the effect was consistently present for DHPs but absent for verapamil, while diltiazem showed mixed results [174,175]. In a study with patients from Scotland, the use of CCB monotherapy (as compared to monotherapy with other antihypertensive drugs) was associated with increased hospital admission rates for mood disorders, mainly major depressive disorder [176]. Similarly, a recent meta-analysis also found an association between the use of CCBs and the risk of depression (albeit very small with an odds ratio of 1.06) [177]. By contrast, in a nationwide study in Denmark, the use of a few antihypertensive drugs, including the CCBs amlodipine and verapamil, was associated with decreased risk of depression [178]. Some studies also looked into the potential role of brain penetrance of the CCBs in distinguishing between non-brain-penetrant DHPs (mainly amlodipine) and brain-penetrant DHPs (most other DHPs including felodipine, isradipine, nifedipine and nitrendipine). Again, results were mixed. One study revealed that brain-penetrant DHPs were associated with a reduced incidence of several psychiatric disorders (e.g., schizophrenia, affective disorder, major depressive disorder) when compared with amlodipine [179]. By contrast, another study did not find an association between the type of DHP (brain-penetrant versus non-brain-penetrant) and the risk of incident psychiatric disorder (schizophrenia, schizoaffective disorder, major depressive disorder, bipolar disorder) [180]. Thus, these mixed results argue against the notion that CCBs might be suitable as a general treatment for a broad range of psychiatric disorders. On the other hand, they do not rule out the possibility that novel, more selective CCBs might be of considerable value for treating certain psychiatric disorders. Afterall, currently available CCBs are neither selective for *CACNA1C*/Ca_V_1.2 nor do they act on specific brain circuits. It is most likely, that a multitude of factors may decide about the potential beneficial effects of a given CCB in a given patient including (but not limited to) the class of the CCB used (DHPs, phenylalkylamines (verapamil), benzothiazepines (diltiazem), or novel, more selective CCBs to be developed) and its pharmacokinetics (e.g., crossing of the BBB), genetic background of the patient (e.g., *CACNA1C* variant present), type of neuropsychiatric disorder, and comorbidities present. Clearly, a better understanding of these factors is mandatory in order to identify patients in whom certain CCBs could be used to treat certain neuropsychiatric disorders successfully, but also to identify patients in whom certain CCBs should be avoided because they might be harmful.

## 6. Conclusions

*CACNA1C*/Ca_V_1.2 acts as a voltage-gated LTCC in cardiomyocytes and neurons. Its proper function is critical for maintaining normal cardiac and brain physiology. Consequently, dysfunctional *CACNA1C*/Ca_V_1.2 has been implicated in cardiac disease (e.g., arrhythmias), where activation of Ca_V_1.2 channels is directly involved in the action potential of pacemaker cells and cells of the working myocardium, and neuropsychiatric disorders as diverse as schizophrenia, bipolar disorder, major depressive disorder, attention deficit-hyperactivity disorder, and autism spectrum disorder. At the cellular level, in the brain, Ca_V_1.2 links neuronal electrical activity with intracellular signaling, gene transcription, cellular development and circuit plasticity. Recent evidence suggests that Ca_V_1.2 also serves pivotal functions in non-neuronal cells in the brain, including astrocytes and microglia, where it may be involved in maintaining the integrity of the BBB and in regulating microglial activation, respectively. Similarly, Ca_V_1.2 or Ca_V_1.1 have been found in peripheral immune cells. The mode of activation of Ca_V_1.2 in electrically non-excitable cells is still not well-understood and some studies suggest that it may rather act as a signaling hub or by other means.

Rodent models with global heterozygous or cell-type- or region-specific knockout of *Cacna1c* represent valuable tools to study the specific roles of Ca_V_1.2 in heart and brain function as well as its potential involvement in cardiac and neuropsychiatric disorders. Behavioral studies in *Cacna1c^+/−^* mouse and rat models have revealed, in part, age- and sex-dependent alterations in socio-affective communication and emotional processing (including anxiety-like behavior) as well as more subtle cognitive alterations. The cellular neuronal mechanisms underlying these behavioral alterations, however, often remain elusive. Moreover, the cardiac phenotype of the same models indicates altered cardiac stress responses, lending support to the general notion that altered expression and/or function of *CACNA1C*/Ca_V_1.2 is sufficient to cause both cardiac and behavioral/neuropsychiatric abnormalities. However, whether—and to what extent—these findings can be translated to human pathophysiology remains an open question.

Currently available LTCC blockers (mainly DHPs, diltiazem, verapamil), which are used clinically to treat cardiovascular disease (hypertension, CAD, arrhythmias), have been evaluated for their potential usefulness in patients with neuropsychiatric disorders with mixed results. The available LTCC blockers, however, are neither selective for Ca_V_1.2, nor do they act on specific organs or brain circuits. More selective Ca_V_1.2-modulating drugs may be required for this purpose. In addition, large, randomized, placebo-controlled, prospective clinical trials are needed to provide more robust evidence for—or against—the usefulness and efficacy of a certain calcium channel blocker (or modulator) in a certain neuropsychiatric disorder and patient.

## Data Availability

No new data were created or analyzed in this study. Data sharing is not applicable to this article.

## Abbreviations

The following abbreviations are used in this manuscript:

AAD	Anti-arrhythmic drug
AF	Atrial fibrillation
AP	Action potential
AV	Atrioventricular
BBB	Blood–brain barrier
*CACNA1C*	Gene encoding the α_1C_ subunit of the Ca_V_1.2 channel
CAD	Coronary artery disease
CaM	Calmodulin
CCB	Calcium channel blocker
CICR	Calcium-induced calcium release
CNS	Central nervous system
CREB	Cyclic adenosine monophosphate response element-binding protein
DHP	Dihydropyridine(s)
ERK	Extracellular signal-regulated kinase
GWAS	Genome-wide association studies
HF	Heart failure
LQT	Long QT syndrome
LTCC	L-type calcium channel(s)
MAPK	Mitogen-activated protein kinase
PKA	Protein kinase A
RyR	Ryanodine receptor
SA	Sinoatrial
SNPs	Single-nucleotide polymorphisms
TS	Timothy syndrome
USV	Ultrasonic vocalization
